# The amino acid content in the daily diet of seniors negatively correlates with the degree of platelet aggregation in a sex- and agonist-specific manner

**DOI:** 10.18632/aging.204229

**Published:** 2022-08-19

**Authors:** Kamil Karolczak, Agnieszka Guligowska, Joanna Kostanek, Bartlomiej Soltysik, Tomasz Kostka, Cezary Watala

**Affiliations:** 1 Department of Haemostatic Disorders, Medical University of Lodz, Lodz, Poland; 2 Department of Geriatrics, Healthy Aging Research Center (HARC), Medical University of Lodz, Lodz, Poland

**Keywords:** diet, amino, acids, sex, platelet aggregability, aging

## Abstract

Aging is a significant risk factor for the development of thrombotic diseases, dependent on blood platelet reactivity. However, the risk of thrombosis also appears to be significantly modulated by dietary nutrient content.

The aim of the current study was to assess the relationship between the amount of amino acids present in the daily diet (not supplemented) and the reactivity of blood platelets to arachidonate, collagen and ADP in 246 women and men aged 60–65 years.

Platelet reactivity was tested using whole blood impedance aggregometry. Amino acid intake was assessed with a 24-hour Recall Questionnaire and calculated with Dieta 5.0 software.

Older subjects receiving higher amounts of all essential amino acids with their daily diet exhibit significantly lower platelet responsiveness to AA-, COL- and ADP in a sex-specific manner: dietary amino acid content was more closely associated with AA- and, to some extent, ADP-induced platelet reactivity in women, and with COL-induced platelet aggregability in men. Therefore, dietary amino acid content may be a novel factor responsible for attenuating platelet reactivity in a sex- and agonist-specific manner.

## INTRODUCTION

A healthy diet helps to reduce unfavorable signs of aging [1]. Nevertheless, the associations between diet pattern and different aspects of aging remain unclear, especially with regard to the prophylaxis of age-related diseases, including thrombotic diseases [2] and cardiovascular aging. While the latter is well recognized as dependent on diet [3], the influence of diet on its key factor, primary platelet haemostasis, remains particularly underinvestigated.

Thrombotic diseases are highly dependent on lifestyle, including nutritional habits [4], and are more likely to manifest in older people [5]. This group of diseases are expected to become more prevalent with the predicted rise in life expectancy and an aging population [6]. Thus, it is important to understand the possible effect of nutrients on the reactivity of platelets in older people, since diet intervention may hopefully become an efficient way to inhibit age-associated platelet activation [7].

The aggregability of blood platelets increases with natural senescence [8] and can be even more aggravated by diabetes and dyslipidemia – metabolic disturbances quite characteristic of older subjects [9–12]. In turn, their presence is strongly dependent on the responsiveness of blood platelets to agonists like arachidonic acid (AA), collagen (COL) or ADP [13–15]. The potential of lifestyle factors, like diet, to reduce platelet aggregability (responsiveness to agonists) in older people and thus attenuate the risk of thrombotic diseases [16–18] remains to be investigated.

In many ways, platelet function is known to be prone to modulation by different food ingredients, such as caffeine or lycopene [19, 20]. However, most substances have only been examined *in vitro* and only a few are so well characterized *in vitro* and *in vivo* [21]. In most cases, these experiments are focused on certain plant secondary metabolites, such as like quercetin, allicin, sulphorafan or resveratrol, which may reflect possible haemostatic effects of plant-rich diets used in some regions of the world where a given type of food/plant medicinal extract/spice is popular [22]; however, such dietary components will clearly vary according to geographical regions. Surprisingly, more common and basic ingredients, such as amino acids, have been neglected by researchers in studies of the potential impact on cardiovascular aging, and particularly on aging-dependent platelet reactivity. Moreover, the vast number of the mentioned studies on plant secondary metabolites and other diet-derived molecules, should be treated with great caution by geriatricians, since many do not use older subjects nor blood platelets taken from older people or animals.

Therefore, our present study offers two key novelties: the first is that it focuses on the more basic constituents of the diet, like amino acids, as the modulators of platelet-dependent haemostasis, and the second is that it includes the blood platelets circulating in the vessels of older subjects. Our work is the first to examine the impact of amino acids on cardiovascular aging and cardiovascular/thrombotic risk with an emphasis on platelet reactivity and aggregability in older men and women.

It remains unknown whether dietary amino acids can be regarded as molecules modulating platelet reactivity in older subjects. Hence, our present study performs whole blood aggregometry of platelets induced with physiological agonists, i.e., arachidonate, collagen and ADP; it also estimates amino acid intake with food 24 hours before blood sampling using a daily diet questionnaire.

## RESULTS

### Relationship between amino acid content in the diet and platelet reactivity in a group of men and women adjusted for age

In the first approach, Spearman’s rank correlation coefficients were calculated as the measure of associations between the estimated amounts of amino acids in food and platelet reactivity (AUC, A_max_, AUC*A_max_/1000) in the whole group of men and women after adjustment for age.

In this group, significant negative correlations were found between platelet aggregation and the amounts of ingested amino acids. This was true for all of the tested amino acids and for all three measured indices of platelet reactivity: AUC, A_max_ and (AUC*A_max_)/1000. No preference regarding the used platelet agonist was noted, i.e., AA, COL or ADP; in all cases, negative correlations were found between the amounts of amino acids present in the diet and platelet reactivity (Table 1). Somewhat different patterns were noted for male and female subgroups.

**Table 1:** Associations between the amounts of amino acids present in daily diet and the variables describing the aggregability of blood platelets in a whole group of probants after the adjustment for age.

**Amino acid**	**AUC**	**A_max_**	AUC*A_max_/1000
Ile [mg]	−0.196^AA #^	−0.151^AA #^	−0.180^AA #^
	−0.197^COL #^	−0.164^COL #^	−0.187^COL #^
	−0.189^ADP #^	−0.163^ADP #^	−0.171^ADP #^
Leu [mg]	−0.196^AA #^	−0.152^AA #^	−0.182^AA #^
	−0.187^COL #^	−0.157^COL #^	−0.178^COL #^
	−0.176^ADP #^	−0.152^ADP #^	−0.160^ADP #^
Lys [mg]	−0.175^AA #^	−0.135^AA #^	−0.158^AA #^
	−0.181^COL #^	−0.147^COL #^	−0.172^COL #^
	−0.185^ADP #^	−0.160^ADP #^	−0.168^ADP #^
Met [mg]	−0.188^AA #^	−0.147^AA #^	−0.173^AA #^
	−0.178^COL #^	−0.145^COL#^	−0.168^COL #^
	−0.176^ADP #^	−0.155^ADP #^	−0.163^ADP #^
Cys [mg]	−0.234^AA #^	−0.200^AA #^	−0.228^AA #^
	−0.158^COL #^	−0.148^COL #^	−0.156^COL #^
	−0.195^ADP #^	−0.178^ADP #^	−0.186^ADP #^
Phe [mg]	−0.192^AA #^	−0.147^AA #^	−0.179^AA #^
	−0.192^COL #^	−0.169^COL #^	−0.187^COL #^
	−0.181^ADP #^	−0.163^ADP #^	−0.169^ADP #^
Tyr [mg]	−0.168^AA #^	−0.114^AA #^	−0.148^AA #^
	−0.179^COL #^	−0.145^COL #^	−0.169^COL #^
	−0.166^ADP #^	−0.141^ADP #^	−0.149^ADP #^
Thr [mg]	−0.175^AA #^	−0.134^AA #^	−0.160^AA #^
	−0.185^COL #^	−0.153^COL #^	−0.176^COL #^
	−0.174^ADP #^	−0.164^ADP #^	−0.168^ADP #^
Trp [mg]	−0.178^AA #^	−0.141^AA #^	−0.163^AA #^
	−0.186^COL #^	−0.150^COL #^	−0.175^COL #^
	−0.180^ADP #^	−0.156^ADP #^	−0.166^ADP #^
Val [mg]	−0.186^AA #^	−0.141^AA #^	−0.170^AA #^
	−0.189^COL #^	−0.156^COL #^	−0.179^COL #^
	−0.181^ADP #^	−0.160^ADP #^	−0.168^ADP #^
Arg [mg]	−0.135^AA #^	−0.102^AA #^	−0.122^AA #^
	−0.143^COL #^	−0.114^COL #^	−0.132^COL #^
	−0.160^ADP #^	−0.139^ADP #^	−0.145^ADP #^
His [mg]	−0.203^AA #^	−0.162^AA #^	−0.187^AA #^
	−0.180^COL #^	−0.141^COL #^	−0.167^COL #^
	−0.198^ADP #^	−0.166^ADP #^	−0.177^ADP #^
Ala [mg]	−0.211^AA #^	−0.168^AA #^	−0.194^AA #^
	−0.168^COL #^	−0.134^COL #^	−0.158^COL #^
	−0.181^ADP #^	−0.159^ADP #^	−0.167^ADP #^
Asp [mg]	−0.219^AA #^	−0.187^AA #^	−0.207^AA #^
	−0.187^COL #^	−0.157^COL #^	−0.179^COL #^
	−0.188^ADP #^	−0.179^ADP #^	−0.185^ADP #^
Glu [mg]	−0.194^AA #^	−0.145^AA #^	−0.178^AA #^
	−0.169^COL #^	−0.145^COL #^	−0.162^COL #^
	−0.166^ADP #^	−0.141^ADP #^	−0.150^ADP #^
Gly [mg]	−0.209^AA #^	−0.170^AA #^	−0.193^AA #^
	−0.161^COL #^	−0.127^COL #^	−0.149^COL #^
	−0.194^ADP #^	−0.179^ADP #^	−0.186^ADP #^
Pro [mg]	−0.194^AA #^	−0.127^AA #^	−0.171^AA #^
	−0.175^COL #^	−0.156^COL #^	−0.170^COL #^
	−0.175^ADP #^	−0.141^ADP #^	−0.152^ADP #^
Ser [mg]	−0.174^AA #^	−0.131^AA #^	−0.159^AA #^
	−0.179^COL #^	−0.151^COL #^	−0.171^COL #^
	−0.155^ADP #^	−0.134^ADP #^	−0.143^ADP #^

Analysis of correlation between the contents of amino acids in the diet and platelet reactivity after the adjustment for age. Results shown as the Spearman’s rank correlation coefficients. Reactivity of blood platelets was measured with impedance aggregometry (see ‘Materials and Methods’) in response to arachidonic acid (AA), collagen (COL) or ADP and recorded as an area under aggregation curve (AUC) or a maximal aggregation (A_max_). These variables were used to calculate (AUC*A_max_)/1000. The amounts of the consumed amino acids ([mg]) represent the levels of amino acid consumption with the diet (without supplements) during the last 24 hours (for details see the section ‘Materials and Methods’). The coefficients of correlations with a statistical significance of at least *P* < 0.05 are marked with ^#^.

### Associations between amino acid content in the diet and platelet reactivity among women

Among women, significant negative associations were found between AA-dependent platelet reactivity and amino acid content, when Spearman’s rank correlations coefficients were adjusted for age (Table 2). For COL-induced aggregability, no significant association was observed (Table 2). For ADP-induced aggregability, a more heterogenous pattern of associations was found between the amounts of amino acids in the diet and the variables describing platelet reactivity; however, these associations always appeared as negative. It appears that older women show lower responsiveness of blood platelets to ADP-stimulation when food and beverages are enriched with Met, His, Ala, Asp, Arg and Gly (Table 2).

**Table 2:** Associations between the amounts of amino acids present in daily diet and the variables describing the aggregability of blood platelets in the subgroup of females after the adjustment for age.

**Amino acid**	**AUC**	**A_max_**	AUC*A_max_/1000
Ile [mg]	−0.274^AA #^	−0.240^AA #^	−0.267^AA #^
	−0.166^COL ns^	−0.129^COL ns^	−0.150^COL ns^
	−0.223^ADP #^	−0.151^ADP ns^	−0.174^ADP ns^
Leu [mg]	−0.266^AA #^	−0.238^AA #^	−0.260^AA #^
	−0.148^COL ns^	−0.110^COL ns^	−0.131^COL ns^
	−0.187^ADP #^	−0.120^ADP ns^	−0.141^ADP ns^
Lys [mg]	−0.258^AA #^	−0.235^AA #^	−0.253^AA #^
	−0.142^COL ns^	−0.118^COL ns^	−0.136^COL ns^
	−0.219^ADP #^	−0.154^ADP ns^	−0.176^ADP ns^
Met [mg]	−0.275^AA #^	−0.251^AA #^	−0.272^AA #^
	−0.132^COL ns^	−0.099^COL ns^	−0.118^COL ns^
	−0.195^ADP #^	−0.135^ADP #^	−0.154^ADP #^
Cys [mg]	−0.281^AA #^	−0.240^AA #^	−0.272^AA #^
	−0.151^COL ns^	−0.107^COL ns^	−0.128^COL ns^
	−0.221^ADP #^	−0.178^ADP ns^	−0.194^ADP ns^
Phe [mg]	−0.266^AA #^	−0.229^AA #^	−0.258^AA #^
	−0.163^COL ns^	−0.126^COL ns^	−0.146^COL ns^
	−0.194^ADP #^	−0.136^ADP ns^	−0.155^ADP ns^
Tyr [mg]	−0.245^AA #^	−0.209^AA #^	−0.237^AA #^
	−0.145^COL ns^	−0.110^COL ns^	−0.131^COL ns^
	−0.177^ADP ns^	−0.111^ADP ns^	−0.131^ADP ns^
Thr [mg]	−0.250^AA #^	−0.214^AA #^	−0.240^AA #^
	−0.140^COL ns^	−0.110^COL ns^	−0.128^COL ns^
	−0.190^ADP #^	−0.148^ADP ns^	−0.163^ADP ns^
Trp [mg]	−0.239^AA #^	−0.211^AA #^	−0.233^AA #^
	−0.138^COL ns^	−0.092^COL ns^	−0.119^COL ns^
	−0.189^ADP #^	−0.124^ADP ns^	−0.149^ADP ns^
Val [mg]	−0.254^AA #^	−0.222^AA #^	−0.248^AA #^
	−0.143^COL ns^	−0.105^COL ns^	−0.128^COL ns^
	−0.176^ADP ns^	−0.118^ADP ns^	−0.137^ADP ns^
Arg [mg]	−0.207^AA #^	−0.179^AA #^	−0.197^AA #^
	−0.093^COL ns^	−0.067^COL ns^	−0.082^COL ns^
	−0.237^ADP #^	−0.168^ADP ns^	−0.193^ADP #^
His [mg]	−0.271^AA #^	−0.242^AA #^	−0.263^AA #^
	−0.143^COL ns^	−0.088^COL ns^	−0.120^COL ns^
	−0.238^ADP #^	−0.154^ADP ns^	−0.182^ADP #^
Ala [mg]	−0.293^AA #^	−0.257^AA #^	−0.283^AA #^
	−0.130^COL ns^	−0.091^COL ns^	−0.113^COL ns^
	−0.215^ADP ##^	−0.161^ADP #^	−0.178^ADP ##^
Asp [mg]	−0.327^AA #^	−0.293^AA #^	−0.318^AA #^
	−0.146^COL ns^	−0.114^COL ns^	−0.132^COL ns^
	−0.203^ADP #^	−0.173^ADP ns^	−0.189^ADP #^
Glu [mg]	−0.229^AA #^	−0.192^AA #^	−0.219^AA ##^
	−0.142^COL ns^	−0.096^COL ns^	−0.120^COL ns^
	−0.151^ADP ns^	−0.087^ADP ns^	−0.106^ADP ns^
Gly [mg]	−0.272^AA #^	−0.228^AA #^	−0.258^AA #^
	−0.111^COL ns^	−0.075^COL ns^	−0.094^COL ns^
	−0.227^ADP #^	−0.183^ADP #^	−0.201^ADP #^
Pro [mg]	−0.253^AA #^	−0.191^AA #^	−0.223^AA #^
	−0.163^COL ns^	−0.128^COL ns^	−0.148^COL ns^
	−0.168^ADP ns^	−0.099^ADP ns^	−0.116^ADP ns^
Ser [mg]	−0.245^AA #^	−0.207^AA #^	−0.236^AA #^
	−0.149^COL ns^	−0.116^COL ns^	−0.134^COL ns^
	−0.159^ADP ns^	−0.104^ADP ns^	−0.124^ADP ns^

Analysis of correlation of the contents of amino acids in the diet and platelet reactivity after the adjustment for age. Results shown as the bootstrap-boosted Spearman’s rank correlation coefficients. Reactivity of blood platelets was measured with impedance aggregometry (see ‘Materials and Methods’) in response to arachidonic acid (AA), collagen (COL) or ADP and recorded as an area under aggregation curve (AUC) or a maximal aggregation (A_max_). These variables were used to calculate (AUC^*^A_max_)/1000. The amounts of the consumed amino acids ([mg]) represent the levels of amino acid consumption with the diet (without supplements) during the last 24 hours (for details see the section ‘Materials and Methods’). The coefficients of correlations with a statistical significance of at least *P* < 0.05 or statistically insignificant are marked with the symbols of ^#^ or ns, respectively. Abbreviations used: AUC, area under aggregation curve; A_max_, maximal aggregation (for details see the section ‘Materials and Methods’).

The female subgroup generally demonstrated less significant changes, where applicable, due to the lower sample size (sample size reduced from 246 to 122 individuals). To confirm the reduction in significance when dividing the sample into subgroups, and to see whether the sample size correction would reverse such a tendency, the subgroup data was analyzed using a bootstrap resampling procedure with an adjustment to the sample size of the overall group (*n* = 246). It was found that the amounts of all of the tested amino acids in the daily diet are negatively and significantly associated with all the hallmarks of blood platelet aggregability: AUC, A_max_ or the overall comprehensive measure of (AUC*A_max_)/1000, when aggregation of female blood platelets is triggered by arachidonate (Table 3).

**Table 3:** Associations between the amounts of amino acids present in daily diet and the variables describing the aggregability of blood platelets in the subgroup of females upon the adjustment for age and for sample size.

**Amino acid**	**AUC**	**A_max_**	AUC*A_max_/1000
Ile [mg]	−0.273^AA †^	−0.240^AA †^	−0.266^AA †^
	−0.164^COL ##^	−0.128^COL #^	−0.150^COL #^
	−0.223^ADP ####^	−0.151^ADP #^	−0.174^ADP ##^
Leu [mg]	−0.266^AA †^	−0.238^AA †^	−0.260^AA †^
	−0.147^COL #^	−0.111^COL *^	−0.131^COL #^
	−0.188^ADP ##^	−0.120^ADP *^	−0.141^ADP #^
Lys [mg]	−0.258^AA †^	−0.235^AA †^	−0.253^AA †^
	−0.142^COL #^	−0.117^COL *^	−0.135^COL #^
	−0.219^ADP ###^	−0.154^ADP #^	−0.176^ADP ##^
Met [mg]	−0.274^AA †^	−0.251^AA †^	−0.271^AA †^
	−0.131^COL ##^	−0.098^COL ns^	−0.118^COL *^
	−0.196^ADP #^	−0.135^ADP #^	−0.154^ADP #^
Cys [mg]	−0.281^AA †^	−0.240^AA †^	−0.272^AA †^
	−0.151^COL #^	−0.107^COL *^	−0.128^COL #^
	−0.222^ADP ###^	−0.179^ADP ##^	−0.194^ADP ##^
Phe [mg]	−0.265^AA †^	−0.229^AA †^	−0.258^AA †^
	−0.163^COL #^	−0.126^COL #^	−0.146^COL #^
	−0.194^ADP ##^	−0.136^ADP #^	−0.155^ADP #^
Tyr [mg]	−0.245^AA †^	−0.209^AA †^	−0.236^AA ###^
	−0.144^COL #^	−0.110^COL *^	−0.131^COL #^
	−0.177^ADP ##^	−0.111^ADP *^	−0.131^ADP #^
Thr [mg]	−0.249^AA †^	−0.214^AA †^	−0.240^AA †^
	−0.140^COL #^	−0.110^COL *^	−0.128^COL #^
	−0.191^ADP ##^	−0.148^ADP #^	−0.163^ADP ##^
Trp [mg]	−0.238^AA ###^	−0.211^AA †^	−0.233^AA †^
	−0.138^COL #^	−0.092^COL ns^	−0.119^COL *^
	−0.190^ADP ##^	−0.124^ADP *^	−0.149^ADP #^
Val [mg]	−0.254^AA †^	−0.222^AA †^	−0.248^AA †^
	−0.143^COL #^	−0.105^COL ns^	−0.128^COL #^
	−0.176^ADP ##^	−0.118^ADP *^	−0.137^ADP #^
Arg [mg]	−0.207^AA ###^	−0.179^AA †^	−0.197^AA ##^
	−0.093^COL ns^	−0.068^COL ns^	−0.082^COL ns^
	−0.238^ADP ###^	−0.169^ADP ##^	−0.193^ADP ##^
His [mg]	−0.270^AA †^	−0.242^AA †^	−0.262^AA †^
	−0.142^COL #^	−0.088^COL ns^	−0.120^COL *^
	−0.239^ADP ###^	−0.154^ADP #^	−0.182^ADP ##^
Ala [mg]	−0.292^AA †^	−0.257^AA †^	−0.282^AA †^
	−0.129^COL #^	−0.091^COL ns^	−0.113^COL *^
	−0.215^ADP ###^	−0.161^ADP #^	−0.178^ADP ##^
Asp [mg]	−0.326^AA †^	−0.293^AA †^	−0.318^AA †^
	−0.146^COL #^	−0.114^COL *^	−0.132^COL #^
	−0.203^ADP ##^	−0.173^ADP ##^	−0.189^ADP ##^
Glu [mg]	−0.228^AA ###^	−0.191^AA †^	−0.219^AA ###^
	−0.141^COL #^	−0.096^COL ns^	−0.120^COL *^
	−0.151^ADP #^	−0.087^ADP ns^	−0.106^ADP^ *
Gly [mg]	−0.272^AA †^	−0.228^AA †^	−0.258^AA †^
	−0.111^COL *^	−0.075^COL ns^	−0.094^COL ns^
	−0.227^ADP ###^	−0.183^ADP ##^	−0.202^ADP ##^
Pro [mg]	−0.235^AA ###^	−0.191^AA †^	−0.223^AA ###^
	−0.163^COL #^	−0.128^COL #^	−0.148^COL #^
	−0.168^ADP ##^	−0.099^ADP ns^	−0.116^ADP *^
Ser [mg]	−0.244^AA †^	−0.206^AA †^	−0.236^AA ###^
	−0.149^COL #^	−0.116^COL *^	−0.134^COL #^
	−0.160^ADP #^	−0.105^ADP ns^	−0.124^ADP ##^

Analysis of correlation of the contents of amino acids in the diet and platelet reactivity after the adjustment for age and for sample size. Results shown as the bootstrap-boosted Spearman’s rank correlation coefficients, estimated by bootstrap resampling with replacement adjusted for the sample size of an overall group (*n* = 246) (10 000 iterations). Reactivity of blood platelets was measured with impedance aggregometry (see ‘Materials and Methods’) in response to arachidonic acid (AA), collagen (COL) or ADP and recorded as an area under aggregation curve (AUC) or a maximal aggregation (A_max_). These variables were used to calculate (AUC*A_max_)/1000. The amounts of the consumed amino acids ([mg]) represent the levels of amino acid consumption with the diet (without supplements) during the last 24 hours (for details see the section ‘Materials and Methods’). The coefficients of correlations with a statistical significance, i.e., *P_2α_* < 0.05, *P_2α_*< 0.01, *P_2α_* < 0.001 or *P_2α_* < 0.0001 are indicated with the symbols of ^#^, ^##^, ^###^ or ^†^, respectively. The statistical tendency (*P* = 0.05 or 0.05 < *P* < 0.1) is indicated with ^*^; the coefficient statistically insignificant are marked as ‘ns’. Abbreviations used: AUC: area under aggregation curve; A_max_, maximal aggregation (for details see the section ‘Materials and Methods’).

The same was found for collagen-stimulated blood platelets, for all amino acids but Arg (no statistical association with AUC, A_max_ or (AUC*A_max_)/1000). Also Gly showed no significant correlations with the overall score of COL-induced platelet reactivity. Some amino acids (Met, Trp, His, Ala, Glu) only demonstrated insignificant negative associations with aggregability (Table 3). Thus, in the female group, Iso, Leu, Lys, Cys, Phe, Tyr, Thr, Val, Asp, Pro and Ser seem to remain the most effective inhibitors of collagen-induced platelet reactivity.

Lastly, in general, the ADP-dependent reactivity of the blood platelets was significantly and negatively associated with amino acid amounts with regard to AUC, A_max_ or (AUC*A_max_/1000). The exceptions were the associations of A_max_ with Glu, Pro and Ser (*p* > 0.05). Some amino acids, including Leu, Tyr, Trp and Val, only demonstrated a tentative relationship with A_max_ (*p* > 0.05); with similar results being noted for Glu and Pro in the case of (AUC*A_max_)/1000 (Table 3).

### Associations between amino acid content in the diet and platelet reactivity in the male subgroup

In the male subgroup, no significant relationship was found between amino acid content in the daily diet and the aggregability of blood platelets induced with either AA, COL or ADP, when adjusted for age (Table 4).

**Table 4:** Associations between the amounts of amino acids present in daily diet and the variables describing the aggregability of blood platelets in the subgroup of males after the adjustment for age.

**Amino acid**	**AUC**	**A_max_**	AUC*A_max_/1000
Ile [mg]	−0.042^AA ns^	0.0150^AA ns^	−0.016^AA ns^
	−0.155^COL ns^	−0.127^COL ns^	−0.150^COL ns^
	−0.071^ADP ns^	−0.075^ADP ns^	−0.082^ADP ns^
Leu [mg]	−0.057^AA ns^	0.004^AA ns^	−0.032^AA ns^
	−0.150^COL ns^	−0.124^COL ns^	−0.148^COL ns^
	−0.072^ADP ns^	−0.075^ADP ns^	−0.080^ADP ns^
Lys [mg]	−0.017^AA ns^	0.038^AA ns^	0.010^AA ns^
	−0.152^COL ns^	−0.109^COL ns^	−0.141^COL ns^
	−0.074^ADP ns^	−0.073^ADP ns^	−0.076^ADP ns^
Met [mg]	−0.028^AA ns^	0.030^AA ns^	0.000^AA ns^
	−0.149^COL ns^	−0.114^COL ns^	−0.141^COL ns^
	−0.069^ADP ns^	−0.069^ADP ns^	−0.076^ADP ns^
Cys [mg]	−0.092^AA ns^	−0.067^AA ns^	−0.091^AA ns^
	−0.074^COL ns^	−0.102^COL ns^	−0.093^COL ns^
	−0.055^ADP ns^	−0.057^ADP ns^	−0.061^ADP ns^
Phe [mg]	−0.034^AA ns^	0.016^AA ns^	−0.015^AA ns^
	−0.135^COL ns^	−0.123^COL ns^	−0.139^COL ns^
	−0.057^ADP ns^	−0.063^ADP ns^	−0.066^ADP ns^
Tyr [mg]	−0.017^AA ns^	0.052^AA ns^	0.014^AA ns^
	−0.134^COL ns^	−0.098^COL ns^	−0.127^COL ns^
	−0.060^ADP ns^	−0.056^ADP ns^	−0.065^ADP ns^
Thr [mg]	−0.018^AA ns^	0.029^AA ns^	0.002^AA ns^
	−0.149^COL ns^	−0.115^COL ns^	−0.143^COL ns^
	−0.058^ADP ns^	−0.060^ADP ns^	−0.065^ADP ns^
Trp [mg]	−0.035^AA ns^	0.009^AA ns^	−0.011^AA ns^
	−0.157^COL ns^	−0.132^COL ns^	−0.152^COL ns^
	−0.079^ADP ns^	−0.077^ADP ns^	−0.087^ADP ns^
Val [mg]	−0.038^AA ns^	0.014^AA ns^	−0.013^AA ns^
	−0.157^COL ns^	−0.121^COL ns^	−0.146^COL ns^
	−0.079^ADP ns^	−0.080^ADP ns^	−0.087^ADP ns^
Arg [mg]	0.021^AA ns^	0.058^AA ns^	0.032^AA ns^
	−0.124^COL ns^	−0.095^COL ns^	−0.117^COL ns^
	0.016^ADP ns^	0.005^ADP ns^	0.008^ADP ns^
His [mg]	−0.055^AA ns^	0.005^AA ns^	−0.024^AA ns^
	−0.144^COL ns^	−0.119^COL ns^	−0.140^COL ns^
	−0.078^ADP ns^	−0.073^ADP ns^	−0.083^ADP ns^
Ala [mg]	−0.052^AA ns^	0.066^AA ns^	−0.025^AA ns^
	−0.142^COL ns^	−0.112^COL ns^	−0.137^COL ns^
	−0.079^ADP ns^	−0.071^ADP ns^	−0.083^ADP ns^
Asp [mg]	−0.037^AA ns^	−0.007^AA ns^	−0.022^AA ns^
	−0.156^COL ns^	−0.130^COL ns^	−0.155^COL ns^
	−0.097^ADP ns^	−0.098^ADP ns^	−0.101^ADP ns^
Glu [mg]	−0.071^AA ns^	−0.011^AA ns^	−0.0478^AA ns^
	−0.107^COL ns^	−0.102^COL ns^	−0.114^COL ns^
	−0.064^ADP ns^	−0.060^ADP ns^	−0.069^ADP ns^
Gly [mg]	−0.062^AA ns^	−0.022^AA ns^	−0.042^AA ns^
	−0.142^COL ns^	−0.116^COL ns^	−0.136^COL ns^
	−0.081^ADP ns^	−0.075^ADP ns^	−0.085^ADP ns^
Pro [mg]	−0.078^AA ns^	0.007^AA ns^	−0.041^AA ns^
	−0.101^COL ns^	−0.091^COL ns^	−0.104^COL ns^
	−0.075^ADP ns^	−0.059^ADP ns^	−0.074^ADP ns^
Ser [mg]	−0.027^AA ns^	0.019^AA ns^	−0.007^AA ns^
	−0.136^COL ns^	−0.114^COL ns^	−0.136^COL ns^
	−0.053^ADP ns^	−0.052^ADP ns^	−0.059^ADP ns^

Analysis of correlation of the contents of amino acids in the diet and platelet reactivity after the adjustment for age. Results shown as the bootstrap-boosted Spearman’s rank correlation coefficients. Reactivity of blood platelets was measured with impedance aggregometry (see ‘Materials and Methods’) in response to arachidonic acid (AA), collagen (COL) or ADP and recorded as an area under aggregation curve (AUC) or a maximal aggregation (A_max_). These variables were used to calculate (AUC*A_max_)/1000. The amounts of the consumed amino acids ([mg]) represent the levels of amino acid consumption with the diet (without supplements) during the last 24 hours (for details see the section ‘Materials and Methods’). The coefficients of correlations with a statistical significance of at least *P* < 0.05 or statistically insignificant are marked with the symbols of # or ns, respectively. Abbreviations used: AUC, area under aggregation curve; A_max_, maximal aggregation (for details see the section ‘Materials and Methods’).

As with the female group, the male subgroup was adjusted to the size of the total group (246 individuals) using bootstrap resampling. In the male subgroup, significant negative correlations, or tendencies, were noted between A_max_, AUC and A_max_*AUC/1000 and amino acids content but only for responsiveness to COL; no such relationships were noted for AA or ADP reactivity. Furthermore, in the case of COL-induced aggregation, the dietary amounts of Cys and Pro showed no relationship with the tested parameters of platelet reactivity (Table 5). Similarly to the female subgroup, these observations remained valid after bootstrap resampling; the procedure did not result in any changes in the observed associations.

**Table 5:** Associations between the amounts of amino acids present in daily diet and the variables describing the aggregability of blood platelets in the subgroup of males after the adjustment for age and for sample size.

**Amino acid**	**AUC**	**A_max_**	AUC*A_max_/1000
Ile [mg]	−0.042^AA ns^	0.015^AA ns^	−0.016^AA ns^
	−0.154^COL #^	−0.126^COL #^	−0.150^COL #^
	−0.071^ADP ns^	−0.075^ADP ns^	−0.080^ADP ns^
Leu [mg]	−0.057^AA ns^	−0.004^AA ns^	−0.032^AA ns^
	−0.150^COL #^	−0.124^COL *^	−0.147^COL #^
	−0.072^ADP ns^	−0.074^ADP ns^	−0.080^ADP ns^
Lys [mg]	−0.017^AA ns^	−0.038^AA ns^	−0.011^AA ns^
	−0.151^COL #^	−0.109^COL *^	−0.140^COL #^
	−0.074^ADP ns^	−0.070^ADP ns^	−0.079^ADP ns^
Met [mg]	−0.028^AA ns^	−0.031^AA ns^	−0.000^AA ns^
	−0.148^COL #^	−0.114^COL *^	−0.141^COL #^
	−0.070^ADP ns^	−0.069^ADP ns^	−0.076^ADP ns^
Cys [mg]	−0.092^AA ns^	−0.066^AA ns^	−0.091^AA ns^
	−0.073^COL ns^	−0.102^COL ns^	−0.092^COL ns^
	−0.055^ADP ns^	−0.057^ADP ns^	−0.061^ADP ns^
Phe [mg]	−0.034^AA ns^	−0.017^AA ns^	−0.015^AA ns^
	−0.135^COL #^	−0.123^COL *^	−0.139^COL #^
	−0.058^ADP ns^	−0.062^ADP ns^	−0.066^ADP ns^
Tyr [mg]	−0.017^AA ns^	−0.052^AA ns^	−0.014^AA ns^
	−0.133^COL #^	−0.097^COL ns^	−0.126^COL #^
	−0.060^ADP ns^	−0.056^ADP ns^	−0.065^ADP ns^
Thr [mg]	−0.017^AA ns^	0.029^AA ns^	−0.002^AA ns^
	−0.148^COL #^	−0.115^COL *^	−0.142^COL #^
	−0.058^ADP ns^	−0.060^ADP ns^	−0.065^ADP ns^
Trp [mg]	−0.035^AA ns^	−0.010^AA ns^	−0.011^AA ns^
	−0.156^COL #^	−0.132^COL #^	−0.154^COL #^
	−0.079^ADP ns^	−0.077^ADP ns^	−0.085^ADP ns^
Val [mg]	−0.038^AA ns^	0.014^AA ns^	−0.012^AA ns^
	−0.147^COL #^	−0.121^COL *^	−0.146^COL #^
	−0.080^ADP ns^	−0.080^ADP ns^	−0.087^ADP ns^
Arg [mg]	−0.020^AA ns^	−0.058^AA ns^	−0.037^AA ns^
	−0.123^COL *^	−0.095^COL ns^	−0.116^COL *^
	0.016^ADP ns^	0.005^ADP ns^	0.008^ADP ns^
His [mg]	−0.054^AA ns^	0.005^AA ns^	−0.024^AA ns^
	−0.014^COL #^	−0.119^COL *^	−0.139^COL #^
	−0.079^ADP ns^	−0.073^ADP ns^	−0.084^ADP ns^
Ala [mg]	−0.052^AA ns^	0.006^AA ns^	−0.025^AA ns^
	−0.141^COL #^	−0.112^COL *^	−0.137^COL #^
	−0.079^ADP ns^	−0.071^ADP ns^	−0.083^ADP ns^
Asp [mg]	−0.037^AA ns^	−0.007^AA ns^	−0.022^AA ns^
	−0.156^COL #^	−0.130^COL #^	−0.155^COL #^
	−0.098^ADP ns^	−0.092^ADP ns^	−0.102^ADP ns^
Glu [mg]	−0.071^AA ns^	−0.010^AA ns^	−0.047^AA ns^
	−0.107^COL *^	−0.102^COL ns^	−0.113^COL *^
	−0.04^ADP ns^	−0.060^ADP ns^	−0.069^ADP ns^
Gly [mg]	−0.062^AA ns^	−0.022^AA ns^	−0.042^AA ns^
	−0.142^COL #^	−0.116^COL *^	−0.136^COL #^
	−0.081^ADP ns^	−0.075^ADP ns^	−0.086^ADP ns^
Pro [mg]	−0.079^AA ns^	0.008^AA ns^	−0.040^AA ns^
	−0.101^COL ns^	−0.091^COL ns^	−0.104^CO ns^
	−0.075^ADP ns^	−0.059^ADP ns^	−0.074^ADP ns^
Ser [mg]	−0.027^AA ns^	0.019^AA ns^	−0.007^AA ns^
	−0.135^COL #^	−0.114^COL *^	−0.135^COL #^
	−0.053^ADP ns^	−0.051^ADP ns^	−0.059^ADP ns^

Analysis of correlation of the contents of amino acids in the diet and platelet reactivity after the adjustment for age and for sample size. Results shown as the bootstrap-boosted Spearman’s rank correlation coefficients, estimated by bootstrap resampling with replacement adjusted for the sample size of an overall group (*n* = 246) (10 000 iterations). Reactivity of blood platelets was measured with impedance aggregometry (see ‘Materials and Methods’) in response to arachidonic acid (AA), collagen (COL) or ADP and recorded as an area under aggregation curve (AUC) or a maximal aggregation (A_max_). These variables were used to calculate (AUC^*^A_max_)/1000. The amount of the consumed amino acids ([mg]) represent the levels of amino acid consumption with the diet (without supplements) during the last 24 hours. (for details see the section ‘Materials and Methods’). The coefficients of correlations with a statistical significance, i.e., *P_2α_* < 0.05, *P_2α_*< 0.01, *P_2α_* < 0.001 or *P_2α_* < 0.0001 are indicated with the symbols of ^#^, ^##^, ^###^ or ^†^, respectively. The statistical tendency (*P* = 0.05 or 0.05 < *P* < 0.1) is indicated with ^*^; the coefficients statistically insignificant are marked as ‘ns’. Abbreviations used: AUC: area under aggregation curve; A_max_, maximal aggregation (for details see the section ‘Materials and Methods’).

### Associations between amino acid content and comprehensive scores of platelet reactivity

In addition to the levels of single amino acids, they were grouped into four main sets for analysis: branched-chain amino acids (valine, leucine, isoleucine), sulfur amino acids (cysteine and methionine), exogenous amino acids (lysine, tryptophan, phenylalanine, threonine, valine, leucine, isoleucine, methionine, arginine and histidine) and endogenous amino acids (cysteine, tyrosine, alanine, aspartic acid, glutamic acid, glycine, proline, serine). The mean intakes of these groups were associated with the markers of platelet reactivity in the whole group of the studied individuals (*n* = 246), as well as in the separate subgroups of men and women.

The results indicate that all of the tested markers of platelet reactivity, i.e., AUC, A_max_ and (AUC*A_max_/1000), were significantly and negatively associated with daily intakes of branched-chain, sulfur, exogenous and endogenous amino acids (Supplementary Table 1).

Following this, the comprehensive scores (CS), a marker of total reactivity of blood platelets, showing ‘summarized’ or ‘global’ reactivity to all of the tested platelet agonists (arachidonate, collagen, ADP) in one common variable, were calculated. The results indicate that ‘global’ AUC, A_max_ and (AUC*A_max_/1000) also demonstrate significant and negative correlations with daily intakes of branched-chain, sulfur, exogenous and endogenous amino acids in older men and women (Supplementary Table 1).

In the examined subgroup of older women, the daily intakes of branched-chain, sulfur, exogenous and endogenous amino acids remained negatively and significantly associated with ‘global’ platelet reactivity; however, the relationships with CS_total_Amax_ and CS_exo_ remained insignificant. The same associations were observed for the original subgroup of 122 women and when the data was adjusted to the size of full group (*n* = 246) (Supplementary Table 2). In contrast, no statistically significant associations were found between the hallmarks of the ‘global’ platelet reactivity and the daily intakes of any of the tested amino acid groups in the male subgroup, either for the original subgroup (*n* = 125) or after adjustment to *n* = 246. These relationships remained maintained (negative significant associations in women and no significant associations in men) also upon the adjustment of the discussed relationships to the set of confounders including several non-collinear blood morphology and plasma biochemistry variables of acceptable tolerance and showing significant differences between sexes: concentration of haemoglobin (HGB), plateletcrit (PCT), platelet-large cells ratio (P-LCR), white blood cell count (WBC), glucose, total cholesterol, high density lipoproteins (HDL), uric acid, animal protein, plant protein and the amount of energy derived of protein (Supplementary Table 2).

Interestingly, the comprehensive scores calculated for branched-chain, sulfur, exogenous and endogenous amino acids were not correlated with the age of the studied male and female probants (the mixed group of *n* = 246); however, covariance analysis found all to be positively and significantly associated with male sex (*P* = 0.00021, *P* = 0.00003, *P* = 0.0001 and *P* = 0.00006 for branched-chain, sulfur, exogenous and endogenous amino acids, respectively). While the ‘global’ intake of branched-chain, sulfur, exogenous or endogenous amino acids was found to be higher in men than women, the ‘global’ platelet reactivity was found to be higher in women than in men (Supplementary Table 3).

In the female subgroup, the total protein content in the diet significantly and negatively correlated with the reactivity of blood platelets to collagen or ADP, but not to arachidonate. However, no such relationship was noted for males. Some relationships were observed for amount of protein per kg of body mass and the content of animal protein in the diet. Neither the amount of plant protein in the diet, nor the amount of energy derived from protein, nor the protein density showed any statistically significant association with any of the tested hallmarks of blood platelet reactivity in all the tested sex groups (Supplementary Table 4).

## DISCUSSION

Aging-related atherogenesis is directly associated with a plethora of molecular mechanisms resulting in the growth of atherosclerotic plaque and poor oxygenation of target tissues [23]. Despite the acquired general knowledge on the role of blood platelets in thrombus growth and atherogenesis, the more detailed mechanisms underlying platelet activation in elderly are poorly investigated, and prophylactic measures based on lifestyle interventions, including diet, remain poorly developed.

Little research has been performed on the influence of diet-derived amino acids on cardiovascular aging, especially with respect to blood platelet reactivity. Hence, our present study examines the relationships between daily consumption of amino acids and blood platelet reactivity in older subjects.

Our findings demonstrate that the amount of amino acids consumed as part of a standard daily diet correlates negatively with blood platelet aggregability, as triggered by physiological agonists, i.e., arachidonate, collagen and ADP. More simply, a higher intake of amino acids is associated with lower blood platelet reactivity. As such, it appears that diet ‘fortification’ by amino acids may reduce the risk of platelet-dependent thrombosis among older women and men. However, the present study is only an initial investigation, and longer observations are needed, ideally supported by *in vitro* and animal models to confirm this.

Our findings indicate that the dietary intake of amino acids is associated with lower reactivity of blood platelets to arachidonate. This implies that in subjects eating food rich in amino acids, the platelets may exhibit a lower risk of acetylsalicylic acid resistance, one of the most popular antiplatelet drugs used in primary and secondary prevention of cardiovascular events; the drug inhibits the metabolism of arachidonic acid by direct inhibition of platelet cycloxygenase-1 [24]. In some cases, like chronic hyperglycaemia [25] or hyperhomocysteinaemia [26], blood platelets demonstrate a lower response to acetylsalicylic acid, with weaker drug effects [27]. Our results open an intriguing question regarding whether diet-derived amino acids can modulate blood platelet sensitivity to acetylsalicylic acid.

*Per analogiam*, the above presented outcomes demonstrating that the ADP-stimulated aggregation of blood platelets is lower in subjects with higher daily intake of amino acids might be perceived in the light of the possible sensitization of platelets resistant to inhibitory action of thienopyridines, acting through irreversible blockade of platelet P2Y_12_ receptor for ADP [28].

In addition, collagen-stimulated aggregation of blood platelets was found to be lower in subjects consuming higher levels of amino acids. This implies that such consumption may block the initial steps of platelet activation, including the adhesion to subendothelial matrix proteins [29, 30].

The lower reactivity of blood platelets observed in older men and women eating higher amounts of essential amino acids may possibly decrease the risk of cardiovascular disease and cardiovascular mortality. Indeed, it has been shown that higher ingestion of proteins, being reservoirs of amino acids, significantly affects both the all-cause and cardiovascular mortality. It has been documented, however, that such an association are specific to the source of proteins: only the ingestion of plant-derived proteins reduces both all-cause and cardiovascular mortality, while the consumption of animal-derived proteins is more associated with the latter [31]. The benefits of plant proteins in regard to cardiovascular mortality have been also observed in another study [32]; this partially confirms our present findings, as they do not distinguish between animal and plant-derived proteins.

Despite this, the content of animal protein in the daily diet was found to correlate significantly and negatively with collagen- and ADP-induced platelet aggregability in women; however, the amounts of plant-derived proteins did not demonstrate any significant relationship with any variables describing blood platelet reactivity, regardless of the tested subpopulation. In future studies, such a differentiation between animal- and plant-derived amino acids would be very valuable, more even so considering that some recently published studies clearly suggest that, unlike plant-derived proteins, meat-derived proteins are associated with shorter longevity, especially in subjects with cardiovascular history, type 2 diabetes or cancer [33].

Occasional reports suggest that some essential amino acids inhibit platelet reactivity. Glycine has been found to suppress blood platelet aggregability *in vivo* and *in vitro*. Its inhibitory action appeared dependent on the presence of the glycine receptor in blood platelets [34]. It is possible that the negative associations found between daily glycine consumption and lower platelet reactivity were derived from the direct interaction of glycine with its blood platelet receptor [34]. In a previous study, in volunteers taking 3 g of histidine for seven days, platelet aggregation and thromboxane production significantly decreased, whereas plasma histidine concentration increased 1.3 times [35]; this is in line with the negative association between platelet reactivity and the concentration of histidine in a daily diet identified herein.

Special note is merited by the branched-chain amino acids. Leucine, isoleucine and valine have been reported to increase platelet activation via a pathway dependent on propionylation of tropomodulin-3 in platelets [36]; this has been associated with an enhanced risk of thrombosis in obese and diabetic subjects [37]. Hence, these three branched-chain amino acids manifest atherogenic effects. This contradicts our present findings, where higher leucine, isoleucine and valine ingestion has associated with lower platelet reactivity in older subjects. It needs to be emphasized that the considerable differences in experimental approaches exist between these reports, and their outcomes require further verification.

Prophylaxis of thrombotic diseases caused by platelet overactivation has a great impact on public health. Unfavorable shifts in the eating patterns toward processed, energy-rich but nutrient-poor food have brought serious and cost-generating problems in public health [38]. It would be interesting to verify whether diets with well-balanced concentrations of amino acids, including environmental approaches promoting healthy behaviors, might serve as part of a wider strategy for reducing the rates of preventable diseases associated with platelet reactivity in older people [39]. Certainly, our preliminary findings, revealed herein, need further verification on larger populations.

One prospective research direction is to the examine the potential discrepancies regarding the pro- and antiatherogenic potential of branched-chain amino acids. Since atherogenesis is a multifactorial process [40], future multivariate analyses should attempt to encompass a broad range of risk factors and end-points, including not only biochemical and molecular markers, but also sociological conditions of eating patterns [41]. Such studies should include subpopulations of older individuals, as they may demonstrate different protein and amino acid metabolisms. While the rate of protein synthesis is particularly accelerated in the muscles, which store the vast majority of body proteins, aged muscles may exhibit a decreased response to dietary amino acids, probably due to increased resistance of the amino acid metabolism to insulin [42]. It is still unknown whether the changes noted in muscles influence both platelet-dependent haemostasis or amino acid sensitivity in blood platelets, and hence, whether they are relevant to the risk of thrombosis. However, our preliminary results, presented herein, suggest that this may be the case. Such a potential biochemical connection between dietary amino acids, the muscle metabolism of proteins and cardiovascular system may initially appear quite exotic. Indeed, very little such evidence exists for blood platelets. Despite this, it is known that in older subjects, the insulin resistance of protein metabolism associates a decreased vasodilation dependent on endothelium dysfunctions [43, 44], suggesting that protein metabolism and the functional state of the haemostatic system seem closely interconnected; however, the role of diet in shaping blood platelet function remains to be established in this regard.

What is particularly interesting are the identified sex- and agonist-specific patterns. In the female subgroup, arachidonic acid-dependent platelet aggregation is affected by all of the tested amino acids; however, only some amino acids are related to ADP-dependent reactivity and no amino acids with COL-induced aggregation. After adjusting for sample size by bootstrap transformation, it was found that the dietary amounts of amino acids were quite uniformly negatively associates with AA-, COL- and ADP-dependent platelet reactivity.

In the male group, no apparent relationships were observed between any kind of platelet reactivity and dietary amounts of amino acids; however, the same sample size adjustment indicted that for almost all amino acids, and most of the implemented markers of platelet reactivity (AUC, A_max_, as well as A_max_*AUC), only COL-triggered platelet aggregability was associated with daily amino acid consumption. Unfortunately, the molecular basis of these sex-, agonist- and amino acid-specific patterns remains unclear.

The studied men tended to eat more total protein than the women. This difference was also noted after dividing total protein intake into plant- and animal-derived proteins. Similar results were also noted for amino acid intake, with the men typically consuming significantly more amino acids (Table 1). However, the women tended to acquire a higher percentage of energy from proteins (Table 1). Again, the molecular basis of the relationship between these sex- and agonist-specific differences and their impact on platelet reactivity remain unclear. Our study is the first to examine the possible relationships between diet amino acids and platelet aggregability in older subjects, with the sex- and the agonist-specific pattern of the discovered associations being of particular interest.

The methodology employed in this study shows some advantages. Whole impedance aggregometry allows authentic measurement of platelet reactivity; while this measurement may lack certain physiological factors, it is nevertheless more accurate than platelet-rich plasma or platelets suspended in artificial buffers.

Regarding limitations, the amino acids consumed during the last 24 hrs was only estimated on the basis of a recall questionnaire, and this can result in errors regarding the composition and size of a portion. In addition, some data may be forgotten; however, to prevent this type of error, the patients are carefully instructed to prepare a preliminary list of products that they would eat within 24 hours before their visit. The interview was carried out by a trained dietitian and nutritionist, who did not comment on the menus but tried to identify the dishes and amounts, and whether there were any deviations from a typical day. This approach provides reliable food consumption data and hence a reliable estimate of the amount of amino acids in the diet. It is particularly useful in nutritional studies in the elderly population.

In addition, the group size was relatively small. However, some more advanced statistical tools were used to overcome this.

It should be emphasized that our method only includes the amounts of amino acids actually ingested with food and beverages: it excludes amino acids derived of cellular metabolic releasates.

In summary, the older subjects receiving higher amounts of all essential amino acids (isoleucine, leucine, lysine, methionine, cysteine, phenylalanine, tyrosine, threonine, tryptophane, valine, arginine, histidine, alanine, aspartic acid, glutamic acid, glycine, proline, serine) with their daily diet exhibit significantly lower platelet responsiveness to AA-, COL- and ADP. These relations appear sex-specific, with higher and more significant correlations found in women. Moreover, a closer relationship was found between amino acid consumption and AA- and ADP-induced platelet reactivity in women; in men, only COL-induced platelet aggregability was associated with dietary amino acids.

Some of our analysis found blood platelets to demonstrate greater sensitivity to the modulatory action of amino acids in female subjects, and greater resistance in male subjects. These outcomes may, however, change with age, which plays a key role in platelet activity. Our findings indicate that diet amino acids are indeed novel factors responsible for attenuating platelet reactivity in a sex-, age- and agonist-specific manner.

## MATERIALS AND METHODS

### Chemicals

Arachidonate, equine tendon collagen and ADP were purchased from Chrono-Log Corp. (Havertown, PA, USA). Physiological buffered saline (PBS) was from Avantor Performance Materials Poland S.A. (Gliwice, Poland). For blood sampling we used S-Monovette^®^ blood collection systems (Sarstedt, Nümbrecht, Germany).

### Study design

This study presents the results obtained in subgroups of volunteers participating in the project entitled “The occurrence of oxidative stress and selected risk factors for cardiovascular risk and functional efficiency of older people in the context of workload”, funded by the Central Institute For Labour Protection – National Research Institute (Warsaw, Poland) and supervised by the Clinic of Geriatrics at the Medical University in Lodz (Poland). Participants responded to the announcements offering the participation in the project, which were published on local TV, radio and newspapers. Two basic inclusion criteria were age within the range of 60 to 65 years and the willingness to participate. The recruitment criteria have been described in more detail in an earlier report [45]. The research group included roughly 300 subjects (equal sex proportions), aged from 60 to 65 years (group-matched age distribution). After excluding volunteers currently taking antiplatelet drugs, we achieved a target population of 122 women and 124 men. The general characteristics of the studied group is presented in Supplementary Table 5.

### Blood sampling, isolation of blood plasma, measurements of blood morphology and serum biochemistry

Blood was taken after overnight fasting and 15-minute rest directly before blood donation. Blood was collected by aspiration either to vacuum tubes (Sarstedt, Nümbrecht, Germany) supplemented with 0.105 mol/l buffered sodium citrate (citrate:blood ratio = 1:9, v/v) (for the measurements of platelet activation and reactivity) or to the tubes coated with EDTAK_3_ in the case of samples taken for blood morphology analysis, or to tubes without any anticoagulant when serum was taken for further biochemical determinations. In all cases, blood was collected from a peripheral vein cannulated with an 18-gauge needle.

Blood morphology was measured with a 5-Diff Sysmex XS-1000i haematological analyser (Sysmex, Kobe, Japan), while a DIRUI CS 400 analyser (Dirui, Changchun, China) was used to evaluate serum biochemical parameters. In order to obtain blood serum, whole blood taken to the tubes without an anticoagulant was incubated for 30 minutes at 37°C and centrifuged (2000 × g/15 min./4°C). Supernatant (serum) was aspirated and used in further analyses (basic blood serum biochemistry).

### Whole blood impedance aggregometry

Platelet aggregability was determined with a MultiPlate Analyzer (Dynabayte, Munich, Germany), as described previously [46]. In brief, samples of citrated blood were left a 10-minute blood reposition at 37°C to avoid the interference of artefactual platelet activation caused by aspiration. The 300 μl aliquots of whole blood were mixed with an equal volume of PBS, gently mixed and left at 37°C for three minutes. Each sample was supplemented with the respective agonist: 0.5 mmol/l arachidonate, 1 μg/ml collagen or 10 μmol/l ADP in order to trigger platelet aggregation. The recording of platelet clumping started immediately thereafter and was tracked for 15 minutes. In this paper we employed three measures of blood platelet reactivity in response to the agonists (arachidonate, collagen, ADP). The area under the aggregation curve (AUC) and maximal aggregation (A_max_) were used as two of three characteristic variables describing platelet aggregability. Those two variables give the optimal measure of the extent of platelet aggregation: how high it is when platelets respond maximally, and how long it lasts before disaggregation starts. These two measures together are much more reliable measures of the extent of platelet aggregation than just one. And it is more difficult to justify selecting only one of these variables for the final description of aggregation. The third one was a derivative variable, calculated according to the following formula: (AUC*A_max_)/1000, combining the previous two to get the most complete description of platelet aggregation.

### Amino acid intake

The daily amino acid intake was calculated based on a detailed analysis of the participants' menu. The intake of food and beverages during the preceding day was estimated by qualified investigators (dietitian and nutritionist) on the basis of a 24-h recall questionnaire using a portion size album. This interview was collected from three days, and then the menu that was most representative of the participant's typical diet was taken for further analysis.

The method of interview can be successfully used for dietary evaluation in the group of elderly people [47]. To decrease the risk of errors from dietary recall, participants were asked to prepare a list of food products (including snacks and beverages) they ate on the day before the appointment. Interviewers were instructed not to judge the diet of the respondents. A photo album was used to determine how many grams the eaten portion had. None of the study participants used protein supplements. The nutritional products declared by patients were analyzed with Dieta 5.0 software (The National Food and Nutrition Institute, Warsaw, Poland). This software platform allows calculation of energy intake and nutrients consumption. We estimated the amounts of the following amino acids: isoleucine (Ile), leucine (Leu), lysine (Lys), methionine (Met), cysteine (Cys), phenylalanine (Phe), tyrosine (Tyr), threonine (Thr), tryptophane (Trp), valine (Val), arginine (Arg), histidine (His), alanine (Ala), aspartic acid (Asp), glutamic acid (Glu), glycine (Gly), proline (Pro) and serine (Ser). Amount of the amino acids [mg] represents the level of amino acid consumption with the diet during the last 24 hours.

### Statistical analysis

The continuous data were presented as either mean ± SD or median with interquartile range (from lower [25%] to upper [75%] quartile). In the case of categorical data, they are presented as percentages. Cumulative measures of amino acid intake were calculated based on the van der Waerden normal scores of individual amino acids for the following amino acid groups: branched-chain amino acids, sulfur containing amino acids, exogenous amino acids and endogenous amino acids. There were referred to as the relevant comprehensive scores: CS_BCAA_, CS_sulfur_, CS_exo_ and CS_endo_, respectively. Likewise, the van der Waerden normal scores of blood platelet aggregation, cumulated through the three employed blood platelet agonists, collagen, ADP and arachidonate, were referred to as the overall scores of ‘cumulative blood platelet aggregation’, CS_total_Amax_, CS_total_AUC_ and CS_total_Amax*AUC_.

Data normality was tested with the Shapiro-Wilk W test and homogeneity of variances with Levene’s test. In the case when the assumptions of data normality and variance homogeneity were not violated, the groups were compared with a non-paired t-Student’s test, otherwise, the U-Mann-Whitney test was used. The associations between variables were calculated as either the partial correlation Pearson’s coefficients with adjustment for compounding variables (multiple regression, partial correlation) or as the Spearman’s rank correlation coefficients. For the purpose of comparisons between subgroups, in order to adjust the monitored variables for the confounders, like age, we used the analysis of covariance (ANCOVA). As a standard, due to relatively small sample sizes in the subpopulations studied and in order to ensure the sufficient statistical power of estimated inferences and associations, we employed the technique of resampling bootstrap (with 5,000 or 10,000 iterations) to minimize that the revealed differences/associations could be observed by pure chance. This approach was also used to estimate the associations in sexual subgroups of individuals upon the adjustment of subgroup sizes (*n*_1_ = 122 for women and *n*_2_ = 124 for men) to the sample size of the overall studied population (*n* = 246). In such situations, we refer in the description of the outcomes to the bootstrap-boosted test statistics instead of the classical approach. Statistical analyses were performed using Statistica v.13 (Dell Inc., Tulsa, OH, USA) and Resampling Stats Add-In for Excel v.4 (The Institute for Statistics Education, An Elder Research Company, Arlington, VA, USA).

## Supplementary Materials

Supplementary Tables 1-4

Supplementary Table 5
