# Supplementary material for: The amino acid content in the daily diet of seniors negatively correlates with the degree of platelet aggregation in a sex- and agonist-specific manner
Source: Aging (Albany NY). 2022 Aug 19;14(18):7240–62. doi: 10.18632/aging.204229 (PMC9550244; doi:10.18632/aging.204229)
Supplement: Supplementary Tables 1-4 [file aging-14-204229-s001.pdf]

SUPPLEMENTARY TABLES

Please browse Full Text version to see the data of Supplementary Table 5.

Supplementary Table 1. Associations between the amounts of the groups of amino acids present in daily diet and the variables describing the aggregability of blood platelets in a whole group of probants after the adjustment for age.

Marker of platelet reactivity	Branched-chain amino acids	Sulfur amino acids	Exogenous amino acids	Endogenous amino acids
$AUC_{arachidonic\ acid}$	−0.193##	−0.217###	−0.183##	−0.183##
$A_{max\_arachidonic\ acid}$	−0.148#	−0.177##	−0.141#	−0.141#
$(AUC*A_{max})/1000_{arachidonic\ acid}$	−0.178##	−0.205##	−0.168##	−0.168##
$AUC_{collagen}$	−0.191##	−0.174##	−0.182##	−0.182##
$A_{max\_collagen}$	−0.160#	−0.152#	−0.149#	−0.149#
$(AUC*A_{max})/1000_{collagen}$	−0.182##	−0.168##	−0.173##	−0.173##
AUC_{ADP}	−0.182##	−0.188##	−0.180##	−0.180#
A_{max_ADP}	−0.158#	−0.168##	−0.157#	−0.157#
$(AUC*A_{max})/1000_{\_ADP}$	−0.166##	−0.175##	−0.165##	−0.165##
CS_{AUC}	−0.216###	−0.222###	−0.161#	−0.161#
CS_{Amax}	−0.168##	−0.179##	−0.190###	−0.190###
$CS_{(AUC*Amax)/1000}$	−0.198##	−0.206##	−0.209##	−0.209##

Analysis of correlation between the contents of branched-chain, sulfur, exogenous and endogenous amino acids in the diet and platelet reactivity after the adjustment for age in a whole group of probants (males and females, *N* = 246); the coefficients are presented for A_{max}, AUC or $AUC*A_{max})/1000$ for individual agonists or as the comprehensive scores cumulated through all the used agonists, CS_{Amax}, CS_{AUC}, $CS_{(AUC*Amax)*1000}$ (for detailed description see 'Statistical analysis'). Results shown as Spearman's rank correlation coefficients. Reactivity of blood platelets was measured with impedance aggregometry (see 'Materials and Methods') in response to arachidonic acid (AA), collagen (COL) or ADP and recorded as an area under aggregation curve (AUC) or a maximal aggregation (A_{max}). These two variables were used to calculate $(AUC*A_{max})/1000$. The amounts of the amino acids consumed with the diet ([mg]), without supplements, represent the van der Waerden's normal scores of the summed daily intakes of amino acids belonging to each of the four distinguished sets during the last 24 hours (for details see the section 'Materials and Methods'). The coefficients of correlations with a statistical significance, i.e., $P_{2\alpha} < 0.05$, $P< 0.01$, $P < 0.001$ are indicated with the symbols of #, ## and ###, respectively.

Supplementary Table 2. Associations between the amounts of the groups of amino acids present in daily diet and the variables describing the aggregability of blood platelets in males and females after the adjustment for sample size, as well as for age or for age and the set of morphology/biochemistry/dietary confounders.

Group of amino acids	CS_{AUC}	CS_{Amax}	$CS_{(AUC*Amax)/1000}$
CS_{BCAA}			
age-adjusted	$-0.233^{F,\ \&(122),}/-0.250^{F,\ \&\&(246)}$	$-0.173^{F,\ \#(122)}/-0.190^{F,\ \#\#(246)}$	$-0.197^{F,\ \#(122)}/-0.218^{F,\ \&(246)}$
	$-0.105^{M,\ n.s.(124)}/-0.138^{M,\ \#(246)}$	$-0.063^{M,\ n.s.(124)}/-0.099^{M,\ n.s.(246)}$	$-0.096^{M,\ n.s.(124)}/-0.128^{M,\ \#(246)}$
Confounders-adjusted	$-0.227^{F,\ \#\#(246)/}-0.080^{M,\ n.s.(246)}$	$-0.192^{F,\ n.s(246)}/-0.050^{M,\ n.s.(246)}$	$-0.216^{F,\ \#(246)}/-0.076^{M,\ n.s.(246)}$
CS_{sulfur}			

Age-adjusted	−0.232$^{F, \&(122)}$/−0.275$^{F, \&\&(246)}$	−0.176$^{F, \#(122)}$/−0.211$^{F, \&(246)}$	−0.200$^{F, \#(122)}$/−0.242$^{F, \&\&(246)}$
	−0.106$^{M, n.s.(124)}$/−0.115$^{M, n.s.(246)}$	−0.072$^{M, n.s.(124)}$/−0.092$^{M, n.s.(246)}$	−0.103$^{M, n.s.(124)}$/−0.114$^{M, n.s.(246)}$
Confounders-adjusted	−0.264$^{F, \&(246)}$/−0.024$^{M, n.s.(246)}$	−0.240$^{F, \#(246)}$/−0.018$^{M, n.s.(246)}$	−0.263$^{F, \#\#(246)}$/−0.029$^{M, n.s.(246)}$
**CS$_{exo}$**			
age-adjusted	−0.224$^{F, \&(122)}$/−0.256$^{F, \&\&(246)}$	−0.166$^{F, n.s.(122)}$/−0.195$^{F, \#\#(246)}$	−0.191$^{F, \#(122)}$/−0.224$^{F, \&(246)}$
	−0.093$^{M, n.s.(124)}$/−0.131$^{M, \#(246)}$	−0.051$^{M, n.s.(124)}$/−0.093$^{M, n.s.(246)}$	−0.082$^{M, n.s.(124)}$/−0.121$^{M, n.s.(246)}$
confounders-adjusted	−0.248$^{F, \#\#(246)}$/−0.041$^{M, n.s.(246)}$	−0.215$^{F, \#(246)}$/−0.017$^{M, n.s.(246)}$	−0.240$^{F, \#(246)}$/−0.039$^{M, n.s.(246)}$
**CS$_{endo}$**			
age-adjusted	−0.230$^{F, \&(122)}$/−0.259$^{F, \&\&(246)}$	−0.171$^{F, \#(122)}$/−0.199$^{F, \#\#(246)}$	−0.196$^{F, \#(122)}$/−0.227$^{F, \&(246)}$
	−0.103$^{M, n.s.(124)}$/−0.128$^{M, \#(246)}$	−0.060$^{M, n.s.(124)}$/−0.091$^{M, n.s.(246)}$	−0.094$^{M, n.s.(124)}$/−0.123$^{M, n.s.(246)}$
confounders-adjusted	−0.254$^{F, \#\#(246)}$/−0.034$^{M, n.s.(246)}$	−0.227$^{F, \#(246)}$/−0.009$^{M, n.s.(246)}$	−0.251$^{F, \#\#(246)}$/−0.031$^{M, n.s.(246)}$

Analysis of correlation between the contents of branched-chain (BCAA), sulfur, exogenous (exo) and endogenous (endo) amino acids in the diet and platelet reactivity after the adjustment for age or for age and the set of confounding variables in separated subgroups of males (M) and females (F). The set of confounders used for the adjustment included several non-collinear blood morphology and plasma biochemistry variables of acceptable tolerance and showing significant differences between sexes: concentration of haemoglobin (HGB) [*red blood cell block of variables*], plateletcrit (PCT), platelet-large cells ratio (P-LCR) [*blood platelet block of variables*], white blood cell count (WBC) [*white blood cell block of variables*], glucose, total cholesterol, high density lipoproteins (HDL), uric acid [*plasma biochemistry block of variables*], animal protein, plant protein and amount of energy derived of protein [*diet protein block of variables*]. Analysis were done in male and female subgroups of original sample sizes (n = 122 and n = 124, respectively) and in the male and female subgroups after their additional adjustment for the sample size of a whole cohort of probands (n = 246). Results shown as the bootstrap-boosted Pearson's partial correlation coefficients between comprehensive scores (CS) calculated for the markers of platelet reactivity cumulated through all the used agonists and the amounts of the groups of amino acids with the use of resampling bootstrap procedure (10,000 iterations; for detailed description see 'Statistical analysis'). Reactivity of blood platelets was measured with impedance aggregometry (see 'Materials and Methods') in response to arachidonic acid (AA), collagen (COL) or ADP and recorded as an area under the aggregation curve (AUC) or a maximal aggregation (A_{max}). These variables were further used to calculate (AUC*A_{max})/1000. The amounts of the amino acids consumed with the diet ([mg]) represent the van der Waerden's normal scores of the summed daily intakes of amino acids (without supplements) belonging to each of the four distinguished sets during the last 24 hours (for details see the section 'Materials and Methods'). The coefficients of correlations with *post hoc* probability values of $P < 0.05$ were considered significant; the significance of $P < 0.05$, $P < 0.01$, $P < 0.001$, $P < 0.0001$, $P > 0.05$ are indicated with the upper symbols of $^{\#}$, $^{\#\#}$, $^{\&}$, $^{\&\&}$, n.s., respectively.

Supplementary Table 3. Comparison of comprehensive scores estimated for the intake of groups of amino acids and for the measures of total reactivity of blood platelets in males and females.

Variable	Average comprehensive score for women	Average comprehensive score for men	*P*
CS$_{BCAA}$	−0.703	0.692	0.00016
CS$_{sulfur}$	−0.508	0.499	0.00002
CS$_{exo}$	−2.331	2.293	0.00013
CS$_{endo}$	−1.930	1.899	0.00004
CS$_{Amax}$	0.653	−0.643	0.00003
CS$_{AUC}$	0.712	−0.700	0.000006
CS$_{(Amax*AUC)/1000}$	0.701	−0.690	0.00001

Comparison of the comprehensive scores (CS, for detailed description see the section 'Statistical analysis') calculated for the intakes of amino acids belonging to each of the four distinguished groups and for the markers of total platelet reactivity (cumulated through arachidonate, collagen and ADP) between men and women. Reactivity of blood platelets was measured

with impedance aggregometry (see 'Materials and Methods') in response to arachidonic acid (AA), collagen (COL) or ADP and recorded as an area under an aggregation curve (AUC) or a maximal aggregation (A_{max}). These variables were further used to calculate (AUC*A_{max})/1000. The amounts of the amino acids consumed with the diet ([mg]) represent the van der Waerden's normal scores of the summed daily intakes of amino acids (without supplements) belonging to each of the four distinguished sets during the last 24 hours (for more detailed description see the section 'Materials and Methods'). The comparison made with the use of bootstrap-boosted *t*-Student's test. Abbreviations used: BCAA: branched-chain amino acids; CS: comprehensive score; endo: endogenous amino acids; exo: exogenous amino acids; sulphur: sulfur amino acids.

Supplementary Table 4. Associations between the markers of protein intake with diet and platelet reactivity in male and female probants.

Indices of platelet reactivity	Amount of all eaten protein [mg]	Protein per kg of body weight [mg]	Animal protein [mg]	Plant protein [mg]	Amount of energy derived from protein [%]	Diet nutritional density [g/1000 kcal]
	−0.125 #, M+−F	−0.085 n.s., M+F	−0.05 n.s., M+F	−0.182 ##, M+F	0.097 n.s., M+F	0.015 n.s., M+F
$AUC_{arachidonic\ acid}$	−0.046 n.s., M	−0.086 n.s., M	0.009 n.s., M	−0.152 #,M	0.041 n.s., M	0.041 n.s., M
	−0.127 n.s., F	−0.159 #, F	−0.062 n.s., F	−0.119 n.s., F	0.051 n.s., F	−0.082 n.s., F
	−0.198 #, M+F	−0.076 n.s., M+F	−0.046 n.s., M+F	−0.145 #, M+F	0.061 n.s., M+F	0.022 n.s., M+F
$A_{max\ arachidonic\ acid}$	−0.015 n.s., M	−0.048 n.s., M	0.057 n.s., M	−0.152 #, M	0.033 n.s., M	0.073 n.s., M
	−0.13 n.s., F	−0.158 #, F	−0.085 n.s., F	−0.045 n.s., F	0.012 n.s., F	−0.071 n.s., F
	−0.128 #, M+F	−0.096 n.s., M+F	−0.062 n.s., M+F	−0.176 ##, M+F	0.083 n.s., M+F	0.028 n.s., M+F
(AUC*A_{max})/1000 arachidonic acid	−0.045 n.s., M	−0.093 n.s., M	0.022 n.s., M	−0.172 #, M	0.037 n.s., M	0.067 n.s., M
	−0.138 n.s., F	−0.167 #, F	−0.081 n.s., F	−0.085 n.s., F	0.028 n.s., F	−0.078 n.s., F
	−0.186 ##, M+F	−0.133 #, M+F	−0.174 ##, M+F	−0.076 n.s., M+F	−0.044 n.s., M+F	−0.062 n.s., M+F
$AUC_{collagen}$	−0.104 n.s., M	−0.1 n.s., M	−0.126 n.s., M	0.028 n.s., M	−0.119 n.s., M	−0.138 n.s., M
	−0.179 #, F	−0.182 #, F	−0.15 #, F	−0.08 n.s., F	−0.047 n.s., F	−0.052 n.s., F
	−0.158 ##, M+F	−0.115 #, M+F	−0.143 #, M+F	−0.084 n.s., M+F	−0.019 n.s., M+F	−0.022 n.s., M+F
$A_{max\ collagen}$	−0.061 n.s., M	−0.058 n.s., M	−0.062 n.s., M	−0.002 n.s., M	−0.061 n.s., M	−0.079 n.s., M
	−0.161#, F	−0.18 #, F	−0.45 #, F	−0.051 n.s., F	−0.048 n.s., F	−0.024 n.s., F
	−0.178 ##, M+F	−0.131#, M+F	−0.165 ##, M+F	−0.08 n.s., M+F	0.037 n.s.M+F	−0.047 n.s.,M+F
(AUC*A_{max})/1000 collagen	−0.095 n.s., M	−0.091 n.s., M	−0.102 n.s., M	−0.001 ## M	−0.092 n.s., M	−0112 n.s., M
	−0.171#, F	−0.18 #, F	−0.152 #, F	−0.063 n.s., F	−0.056 n.s., F	−0.05 n.s., F
	−0.172 ##, M+F	−0.128 #, M+F	−0.153 ##, M+F	−0.186 ##, M+F	0.04 n.s., M+F	−0.033 n.s., M+F
AUC_{ADP}	−0.038 n.s., M	−0.051 n.s., M	−0.056 n.s., M	−0.035 n.s., M	−0.073 n.s., M	−0.085 n.s., M
	−0.203 #, F	−0.255 ##, F	−0.174 #, F	−0.184 n.s., F	−0.008 n.s., F	−0.084 n.s., F
	−0.136 #, M+F	−0.094 n.s., M+F	−0.111#, M+F	−0.140 ##, M+F	0.029 n.s., M+F	−0.004 n.s., M+F
$A_{max\ ADP}$	−0.04 n.s., M	−0.052 n.s., M	−0.021 n.s., M	−0.052 n.s., M	−0.052 n.s., M	−0.058 n.s., M
	−0.123 #, F	−0.178#, F	−0.123 #, F	−0.078 n.s., F	−0.013 n.s., F	−0.022 n.s., F
	−0.143 #, M+F	−0.102 n.s., M+F	−0.123 #, M+F	−0.151 n.s., M+F	0.029 n.s., M+F	−0.009 n.s., M+F
(AUC*A_{max})/1000_{ADP}	−0.038 n.s., M	−0.049 n.s., M	−0.038 n.s., M	−0.045 n.s., M	−0.061 n.s., M	−0.075 n.s., M
	−0.143 #, F	−0.197 #, F	−0.131 #, F	−0.104 n.s., F	−0.01 n.s., F	−0.041 n.s., F

Analysis of correlations between the markers of protein intake with diet and platelet reactivity in the combined group of male and female probants (n = 246) and in the separate sexual subgroups of males and females (n = 124 and n = 122, respectively). Results shown as Spearman's rank correlation coefficients. Reactivity of blood platelets was measured with impedance aggregometry (see 'Materials and Methods') in response to arachidonic acid (AA), collagen (COL) or ADP and recorded either as an area under an aggregation curve (AUC) or a maximal aggregation (A_{max}). These two variables were further used to calculate (AUC*A_{max})/1000. Dietetic variables describing protein intake represent the levels of consumption with the diet (without supplements) during the last 24 hours (for details see the section 'Materials and Methods'). The coefficients of correlations with $P < 0.05$, $P < 0.01$ or $P > 0.05$ are indicated with the symbols of $^{\#}$, $^{\#\#}$, or $^{n.s.}$ respectively. Abbreviations used: CS: comprehensive score (for details see also the section 'Statistical analysis'); F: females; M: males.

Supplementary Table 5. Blood morphology, plasma/serum biochemistry, intake of proteins and amino acids, anthropometric, medical history and medical treatment variables reported in the studied group.
