# Supplementary material for: The amino acid content in the daily diet of seniors negatively correlates with the degree of platelet aggregation in a sex- and agonist-specific manner
Source: Aging (Albany NY). 2022 Aug 19;14(18):7240–62. doi: 10.18632/aging.204229 (PMC9550244; doi:10.18632/aging.204229)
Supplement: Supplementary Table 5 [file aging-14-204229-s002.docx]

**Supplementary Table 5. Blood morphology, plasma/serum biochemistry, intake of proteins and amino acids, anthropometric, medical history and medical treatment variables reported in the studied group.**

| **Variable** | **Both sexes (*n* = 246)** | **Males (*n* = 124)** | **Females (*n* = 122)** |
| --- | --- | --- | --- |
| *Indices of blood morphology and biochemistry* |  |  |  |
| WBC (10^3^/mm^3^) | 5.8 (5.0 – 6.9) | 6.0 (5.0 – 6.9) | 5.6 (5.07 – 6.8)^U*^ |
| RBC (10^6^/mm^3^) | 4.5 ± 0.4 | 4.7 (4.4 – 4.9) | 4.3 ± 0.3^T††^ |
| HGB (g/dl) | 13.8 (13.0 – 14.6) | 14.4 (13.7 – 14.9) | 13.3 ± 0.80^U††^ |
| HCT (%) | 39.8 (37.6 – 41.6) | 41.1 (39.2 – 42.6) | 38.5 ± 2.2^U††^ |
| PLT (10^3^/mm^3^) | 213 (181 – 243) | 197 (168.5 – 228.5) | 226.0 ± 44.7^U††^ |
| MPV (µm^3^) | 11.3 (10.8 – 12.1) | 11.2 ± 0.9 | 11.35 ± 1.01 |
| PCT (%) | 0.24 (0.21 – 0.28) | 0.22 (0.2 – 0.23) | 0.26 ± 0.05^U††^ |
| PDW (fl) | 13.6 (12.4 – 15.6) | 13.5 (12.1 – 15.2) | 13.8 (12.7 – 16.3)^U*^ |
| P-LCR (%) | 36.1 ± 7.7 | 35.7 ± 7.4 | 36.5 ± 8.4^T*^ |
| Lym (10^3^/mm^3^) | 2.0 (1.6 – 2.4) | 1.96 (1.5 – 2.2) | 1.94 ± 0.5 |
| Mono (10^3^/mm^3^) | 0.5 (0.5 – 0.7) | 0.57 (0.5 – 0.7) | 0.51 (0.4 – 0.6)^U††^ |
| Neu (10^3^/mm^3^) | 3.1 (2.6 – 3.9) | 3.18 (2.5 – 3.8) | 2.99 (2.5 – 4.0) |
| Eo (10^3^/mm^3^) | 0.2 (0.1 – 0.2) | 0.13 (0.1 – 0.2) | 0.17 (0.09 – 0.2)^U*^ |
| Baso (10^3^/mm^3^) | 0.03 (0.02 – 0.03) | 0.03 (0.02 – 0.03) | 0.03 (0.02 – 0.03) |
| Total cholesterol (mg/dl) | 206.8 (173.8 - 237.3) | 187.2 (168.7 – 218.3) | 223.1 ± 49.9^U††^ |
| Triglycerides (mg/dl) | 111.2 (76.8 – 161.1) | 111.2 (77.4 – 141.3) | 110.5 (78.4 – 164.4) |
| HDL cholesterol (mg/dl) | 48.4 (41.0 – 59.3) | 44.3 (40.2 – 51.0) | 54.25 (44.1 - 63.4)^U††^ |
| LDL cholesterol (mg/dl) | 131.2 (103.4 - 156.5) | 116.3 (101.4 – 139.0) | 140.1 ± 39.6 ^U†^ |
| Glucose (mg/dl) | 99.2 (91.4 – 108.3) | 101.0 (93.8 – 111.6) | 96.35 (89. - 105.4)^U#^ |
| Uric acid (mg/dl) | 4.84 ± 1.24 | 5.40 (4.8 – 6.1) | 4.30 (3.8 – 5.2)^T††^ |
| *Protein and amino acids daily intake* |  |  |  |
| Total protein intake [mg] | 72.2 (54.9 – 85.9) | 77.6 (59.8 – 94.0) | 67.1 (50.0 – 81.4)^U††^ |
| Protein per kg of body mass [mg] | 0.9 (0.7 – 1.2) | 0.9 (0.7 – 1.2) | 0.9 (0.7 – 1.2) |
| Animal protein [mg] | 45.3 (30.8 – 59.1) | 50.2 (36.1 – 62.9) | 42.3 (27.2 – 55.4)^U#^ |
| Plant protein [mg] | 23.8 (17.7 – 30.3) | 25.9 (19.9 – 32.8) | 21.3 (15.9 - 28.5)^U††^ |
| Amount of energy derived from protein [%] | 16.5 (13.5 – 19.7) | 15.8 (13.2 – 18.6) | 17.1 (14.3 – 21.0)^U#^ |
| Protein density [g/1000 kcal] | 41.2 (33.7 – 49.6) | 39.6 (32.9 – 47.0) | 42.6 (35.1 – 52.5)^U††^ |
| Ile [mg] | 3406  (2546; 4155) | 3642  (2814; 4477) | 3196  (2392; 3873)^†^ |
| Leu [mg] | 5348  (4081; 6378) | 5712  (4402; 7052) | 5018  (3808; 5957)^†^ |
| Lys [mg] | 4654  (3435; 5822) | 4994.12  (3741; 6394) | 4406  (3279; 5319)^†^ |
| Met [mg] | 1652  (1245; 2034) | 1772  (1367; 2247) | 1582  (1151; 1 900)^†^ |
| Cys [mg] | 1014  (769; 1269) | 1092  (859; 1396) | 959  (664; 1143)^††^ |
| Phe [mg] | 3127  (2432; 3722) | 3374  (2559; 4194) | 2885  (2141; 3533)^††^ |
| Tyr [mg] | 2539  (1889; 3088) | 2766  (2070; 3211) | 2317  (1727; 2876)^†^ |
| Thr [mg] | 2890  (2177; 3467) | 3079  (2384; 3983) | 2672  (2037; 3253)^†^ |
| Trp [mg] | 899  (670; 1093) | 954  (735; 1191) | 837  (620; 1029)^†^ |
| Val [mg] | 4005  (3066; 4908) | 4228  (3371; 5245) | 3835  (2798; 4594)^†^ |
| Arg [mg] | 3525  (2627; 4434) | 3641  (2899; 4750) | 3276  (2380; 4135)^†^ |
| His [mg] | 1951  (1454; 2507) | 2118  (1580; 2678) | 1782  (1327; 2 335)^†^ |
| Ala [mg] | 3263  (2442; 4215) | 3546  (2662; 4556) | 3148  (2217; 3902)^#^ |
| Asp [mg] | 6305  (4646; 7878) | 6670  (5138; 8871) | 5930  (4394; 7341)^†^ |
| Glu [mg] | 13696  (10795; 16678) | 15117  (11791; 17966) | 12883  (9235; 15125)^††^ |
| Gly [mg] | 2913  (2106; 3792) | 3125  (2347; 4134) | 2721  (1960; 3423)^†^ |
| Pro [mg] | 5051  (3718; 6200) | 5422  (4221; 6615) | 4589  (3356; 5760)^††^ |
| Ser [mg] | 3398  (2602; 4054) | 3628  (2787; 4327) | 3133  (2320; 3864)^†^ |
| *Medical indices* |  |  |  |
| Age [years] | 62.4 ± 1.7 | 62.9 ± 1.7 | 62.6 ± 1.6^T*^ |
| Regular physical activity [%] | 70 | 70 | 70 |
| Current smoking | 23 | 24 | 22 |
| BMI [kg/m^2^] | 27.3 (24.7 – 30.3) | 27.5 (25.06 – 30.4) | 27.2 (24.4 – 30.04) |
| WHR [cm] | 0.92 (0.84 – 1) | 0.99 (0.94 – 1.04) | 0.84 (0.80 – 0.91)^††^ |
| Hypertension [%] | 47 | 54 | 39 |
| Hypercholesteroleamia [%] | 63 | 59 | 66 |
| Type 2 diabetes mellitus [%] | 9 | 11 | 7 |
| Myocardial infarction in the past [%] | 2 | 2 | 0,8 |
| Stroke in the past [%] | 2 | 3 | 2 |
| Osteoporosis [%] | 11 | 2 | 20 |
| Diseases of stomach and duodenum [%] | 33 | 27 | 39 |
| Cancer in the past [%] | 7 | 6 | 7 |
| Ophtalmologic diseases [%] | 20 | 19 | 20 |
| Depression [%] | 15 | 10 | 20 |
| Chronic obstructive pulmonary disease [%] | 13 | 11 | 14 |
| Joints diseases [%] | 48 | 44 | 52 |
| *Medicines taken currently* |  |  |  |
| Blocker of histamine receptor H2 [%] | 0 | 0 | 0 |
| Acetylsalicylic acid [%] | 0 | 0 | 0 |
| Clopidogrel or ticlopidine | 0 | 0 | 0 |
| Acenonocoumarol [%] | 0 | 0 | 0 |
| Nitrates [%] | 0.4 | 0.8 | 0 |
| Beta blockers [%] | 19.5 | 19 | 20 |
| Digoxin [%] | 0 | 0 | 0 |
| Angiotensin converting enzyme inhibitors [%] | 18.2 | 18 | 19 |
| Calcium channel blockers [%] | 10.6 | 14 | 7 |
| Indapamide [%] | 11 | 13 | 9 |
| Spironolactone [%] | 0.8 | 0.8 | 0.8 |
| Sartans [%] | 5.7 | 5 | 6.5 |
| Thiazide [%] | 3.2 | 2.4 | 4 |
| Amiloride [%] | 0.4 | 0 | 0.8 |
| Torsemide [%] | 0.4 | 0 | 0.8 |
| Eplerenone [%] | 0 | 0 | 0 |
| Alpha blockers [%] | 5.7 | 11.3 | 0 |
| Statins [%] | 17 | 19 | 15 |
| Fibrates [%] | 2.4 | 0.8 | 4 |
| Bisphosphonates [%] | 2.4 | 0 | 5 |
| Allopurinol [%] | 2.4 | 3.2 | 1.6 |
| Insulin [%] | 2.4 | 5 | 0 |
| Metformin [%] | 6.5 | 6.5 | 6.6 |
| Gliclazide/glimepiride [%] | 3.7 | 5.6 | 1.6 |
| Steroids [%] | 1.6 | 0 | 3.3 |
| Methotrexate [%] | 1.2 | 0.8 | 1.6 |
| Non-steroidal anti-inflammatory drugs [%] | 2 | 2.4 | 1.6 |
| Beta mimetics [%] | 3.2 | 2.4 | 4 |
| Antihistamines [%] | 1.6 | 0 | 3.2 |
| Antidepressants [%] | 3.2 | 1.6 | 5 |
| Neuroleptics [%] | 0.8 | 0.8 | 0.8 |
| Vinpocetine/nootropics [%] | 3.7 | 4 | 3.3 |
| Trimetazidine [%] | 0.8 | 0.8 | 0.8 |
| Mesalazine [%] | 0.4 | 0 | 0.8 |
| Trimebutine [%] | 0.8 | 0.8 | 0.8 |
| Diosmin [%] | 2.4 | 0 | 5 |
| Levodopa [%] | 0.4 | 0.8 | 0 |

Variables (not adjusted) presented as means ± SD, median with interquartile ranges (from lower [25%] to upper [75%] quartile) or percentage fractions of a whole group of investigated patients. The amounts of the consumed amino acids ([mg]) represent the levels of amino acid consumption with the diet (without supplements) during the last 24 hours. Comparisons between men and women performed with the use of unpaired Student *t* test (^T^) or Mann–Whitney *U* test (^U^)^&^.

^*^*P*≤ 0.05

^#^*P*< 0.01

^†^*P*< 0.001

^††^*P*< 0.0001

^&^ As a result of the perfect age-matching of the subpopulations of men and women, the comparisons between men and women performed with the use of the bootstrap-boosted analysis of covariance with the adjustment for age, demonstrated almost the same results (*data not shown*).

*Abbreviations used:* Ala, alanine; Arg, arginine; Asp, aspartic acid; Baso, number of basophils; BMI. body mass index; Cys, cystine; Eo, number of eosinophils; Glu, glutamic acid; Gly, glycine; HCT, haematocrit; HDL, high density lipoproteins; HGB, concentration of haemoglobin; His, histidine; Ile, isoleucine; Leu, leucine; Lys, lysine; LDL, low density lipoproteins; LYM, number of lymphocytes; Met, methionine; Mono, number of monocytes; MPV, mean platelet volume; Neu, number of neutrophils; Phe, phenylalanine; PCT, plateletcrit; PDW, platelet distribution width; P-LCR, platelet-large cells ratio; PLT, platelet count; Pro, proline; RBC, red blood cell count; Ser, serine; Thr, threonine; Trp, tryptophane; Tys, tyrosine; WBC, white blood cell count; WHR, weist-hip ratio; Val, valine.
